# A Case of Acute Ulcerative Endocarditis
1Read before the Bristol Medico-Chirurgical Society, November 9th, 1898.


**Published:** 1899-03

**Authors:** H. L. Ormerod


### A CASE OF ACUTE ULCERATIVE ENDOCARDITIS.[1]

BY

H. L. Ormerod, M.D.R.U.I., M.R.C.S., L.R.C.P.

Cases of ulcerative or malignant endocarditis are always interesting, but my special reasons for bringing this one before the Society are:

(1) Its unusual acuteness.

(2) The persistent pyrexia, without any rigor or marked remission in temperature.

(3) It is another example of the value of Widal's test in cases of, or resembling, enteric fever.

[1] Read before the Bristol Medico-Chirurgical Society, November 9th, 1898.

The patient was a farmer, aged twenty-nine years, of somewhat slight build, who, with the exception of occasional mild rheumatic attacks, none of which were sufficiently severe to induce him to consult a medical man, had always enjoyed good health. There is nothing of interest in the family history.

first saw him on Monday, October 17th, 1898. He then told me that for the past week or ten days he had felt tired, and suffered a good deal from headache and pains in the back and limbs, but had continued his work until the previous day; the bowels had acted regularly each day, and he had not observed anything unusual about the motions. For some days he had been suffering from a small sore on the outer side of the left heel, caused by his boot, but had had no tenderness or enlargement of the lymphatic glands, nor could I get any history of lymphangitis.

When I first saw him he was up and dressed, complaining of general malaise, frontal headache, pain in lower part of the back and limbs, chiefly in the larger joints, and of some slight cough, with rusty-coloured tenacious expectoration. There was no history of shivering. Temperature was 103°, skin just moist, no redness of joints and no effusion into them, but slight tenderness over them and some pain on movement. Respirations about twenty per minute, and no abnormal sounds to be heard in the lungs. There was, however, a loud diastolic aortic murmur, with, so far as I could ascertain, very little if any displacement of the apex-beat. Pulse about 100. Tongue clean and moist, and abdomen apparently normal. I suspected that he was suffering from a small patch of deep-seated pneumonia, and possibly influenza. I ordered him to bed, and treated him with salicylate of sodium, with liquor ammoniæ acetatis and sal volatile.

For the first five days of my attending him he appeared to be progressing satisfactorily; the temperature ranged from 103°—104°, cough and expectoration diminished, the cardiac bruit did not alter in character. The bowels continued to act once each day, the motions being dark-coloured and formed; tongue remained moist and was very little furred; there was still, however, some headache and pains in the joints, especially shoulders, elbows, hips and knees, with tenderness over them, but no effusion; and although I could not detect any abnormal physical signs in the lungs, I saw no reason to change my diagnosis. On the evening of the fifth day patient was a little delirious, and when I saw him on the sixth day (Saturday) was certainly worse. Temperature was 104°, but the pulse and respiration had risen to 120 and 24 respectively; still, there was no alteration in the physical signs of the chest. The tongue was becoming dry; abdomen a little flatulent, with some tenderness over region of the bladder, but no gurgling over cæcum, and no typhoid spots; the spleen extended to edge of the ribs; there was still a little delirium; no deafness, but some slight tinnitus, probably due to the salicylate of sodium, I thought, and the patient answered questions sharply. Pupils equal and of about the normal size, reacted readily to light; skin much more moist; trace of albumen, but no blood in urine, and some tremor of muscles. The patient was lying on his back, but he had done so all through the illness, on account of the pain in the shoulders. The next day (Sunday) pulse had risen to over 140, and respiration to 36; delirium continued, tongue was dryer; three light-coloured loose motions had been passed in the previous twenty-four hours; spleen could be distinctly felt just below edge of ribs; there was no increase of abdominal distension, but slight gurgling over the cæcum; no typhoid spots, but two small patches of petechiæ—one just over the left anterior superior iliac spine, and the other in right hypochondrium; tremor much more marked and subsultus present. Towards night, delirium increased, temperature rose to 105°, and urine was passed unconsciously. At midday next day patient would still answer questions rationally, pulse and respiration 144 and 48 respectively; the aortic diastolic bruit was still the only murmur present; there was some impaired resonance and bronchial breathing at the base of left lung, but no crepitation or sign of any general bronchial catarrh. Dozens of petechiæ over chest, abdomen, back, and especially the extremities, those on the hands and feet being the largest. There was no evidence of any affection of the cranial nerves. Abdomen a little more distended, but no tenderness, and no action of the bowels since the previous morning. The blood did not give the typhoid reaction to Widal's test. The patient gradually became more and more drowsy, general diffuse râles became audible over both lungs; temperature rose steadily to 106.2°, and in spite of 30 grs. of quinine and frequently-repeated doses of phenacetin, together with cold sponging, only fell to 104.6° before death, which took place early next morning, or on the ninth day of my attendance.

At the *post-mortem* examination, made thirty-two hours after death, in which Dr. J. O. Symes kindly assisted me, the heart was found to be a little enlarged, the left ventricle being hypertrophied. The aortic valves, which were incompetent and also considerably thickened and puckered, had several small vegetations upon them and also some ulceration; there were also two small patches of lymph upon, and slight ulceration of, the endocardium of the left ventricle, otherwise the heart appeared normal. There were petechial patches over the visceral pleura; but the lungs, with the exception of considerable engorgement and *post-mortem* changes, were normal. Spleen was about twice the natural size, studded with small infarcts; the kidneys also were dotted with petechiæ and a few infarcts; the intestines normal; liver normal. We could not obtain leave to examine the brain. Cultures were subsequently made from the infarcts by Dr. Symes, and found to consist of pure streptococci; and in sections of the aortic valves, kindly made for me by Mr. A. L. Flemming, are to be seen streptococci and staphylococci.

The complete absence of any rigor or of marked remissions in the temperature, which are so characteristic of ulcerative endocarditis, made the nature of the case somewhat uncertain; indeed, until after the fifth day, I saw no reason to doubt the original diagnosis of a small deep-seated pneumonia. At about this date, however, the enlarged spleen and typhoidal condition of the patient led me to strongly suspect enteric fever, especially with the history of an indefinite onset of about ten days, followed by the continued pyrexia; still the rapid downward progress of the case, with the absence of deafness, bronchial catarrh, or definite intestinal symptoms, with the exception of the loose motions on one day, combined with the clean tongue and mental acuteness of the patient, seemed strongly against this view, and pointed rather to a form of septicæmia, and my opinion was subsequently strengthened by the absence of the Widal reaction. With the exception of the small sore on the foot previously referred to, and which did not appear to have caused any constitutional symptoms, I could discover no external source, and was thus led to a diagnosis of ulcerative endocarditis of the acute typhoidal type. I regret, however, that no cultures were made from the blood before death.

These cases are quite different in their clinical symptoms from the more common forms of ulcerative endocarditis, namely, the subacute and chronic cases, and are especially severe and fatal, the patient usually being seized with a rigor when apparently in good health, or convalescent from an acute illness, especially rheumatism, and are said by Mitchell Bruce to always result in death within fourteen days; in fact, a case has been known to terminate fatally in two days. The failure of every means of cure hitherto employed in these acute cases serves to impress on us the importance of preventive treatment; to protect subjects with chronic valvular disease against zymotic diseases, and also attend with special care to the treatment of suppuration. When ulceration has occurred, the case must be managed on the general principles applicable to all acute specific fevers. Drugs in great variety have been tried with little benefit, including mercurials, quinine, and other antipyretics, eucalyptus, turpentine, salicylates, and sulphocarbolates. The two latter have, however, been attended with success in a few instances in the chronic form of the disease. During the past few months some cases have been treated with injections of the anti-streptococcus serum, and although some good may very possibly be done in the subacute or chronic cases, so far as I can find, no acute case has been successfully treated by this means, and, indeed, from the rapidly fatal issue of these cases, so powerful a dose of the serum would be required that the percentage of recoveries by this means of treatment would be extremely small.

In conclusion, in addition to Dr. Symes and Mr. Flemming for pathological help, I must thank Dr. Shingleton Smith for clinical assistance in the case.

In the discussion which followed the reading of Dr. Ormerod's case, Dr. SHINGLETON SMITH commented on the rarity of these cases of acute fulminating septic endocarditis. He alluded to the difficulty of differentiating them from enteric fever, and to the utility of the Widal test for this purpose. In allusion to the streptococcic serum, he stated that his experience of it in a fatal case of pemphigus foliaceus had not been favourable.—Dr. WALDO said this case bears out the view that when pneumonia is associated with ulcerative endocarditis the latter is of an unusually malignant type. There are present in this condition all the signs of septic infection, and, as Wilks has well said, this disease should be regarded as one of arterial pyæmia. The treatment, in his experience, was always unfavourable. He had administered intestinal antiseptics of various kinds, and had given perchloride of mercury hypodermically, but, he must confess, with very little good result.—Dr. B. M. H. ROGERS showed a heart with large vegetations on two of the aortic valves, which was taken from the body of a gipsy girl, aged 11, who died in the Children's Hospital, after only three days' illness. She was taken ill on a Thursday, and when admitted to the Hospital was practically moribund. A loud murmur was heard over the base of the heart, its loudest spot being over the pulmonary artery. At the necropsy the left ventricle wall was found to be much hypertrophied, and two vegetations were present on the aortic valves. There were no infarcts found anywhere. For some months previous to her fatal illness, her parents had noticed that her abdomen was much enlarged, and at the necropsy this was ascertained to be due to a much enlarged spleen. The substance of this organ was very hard, not friable as is usually met with in cases of malignant endocarditis. He mentioned this case to illustrate the exceedingly rapidly fatal issue that may follow infection of this sort, death resulting after only three days' illness.—Dr. G. PARKER said that in a case of ulcerative endocarditis recently under his care the patient's blood showed the presence of staphylococcus albus, but no serum at present existed which had any effect upon this organism. He had used the streptococcal serum as well as quinine, and injections of corrosive sublimate, without influencing the course of the disease.—Dr. J. O. SYMES said that the organism in Dr. Ormerod's case proved on cultivation to be the streptococcus longus, and colonies of the same were found in sections of the valves. The blood did not give Widal's reaction. In another case, with well-marked vegetations on the valves, no organisms could be found in the tissues after death. He did not regard this as a true infective endocarditis. For purposes of diagnosis, two or three cubic centimetres of blood should be taken from a vein and cultures made. Treatment by anti-streptococcus serum was a specific treatment, and useless against other infections.—Dr. WALTER SWAYNE said it was important to know if those who had used the anti-streptococcic serum largely had noticed any ill effects to follow; it was generally stated that ill effects were not experienced, and, that being so, it would appear that the empirical use of the serum was not only justifiable, but to be recommended, since the difficulty of identifying scientifically a true case of streptococcic infection was not only great but required time which might be of the greatest value. He would feel inclined to use the serum in suspected cases without waiting for bacteriological verification of the true source of the poisoning, although such a course might be said to be scientifically incorrect. —Dr. F. H. Edgeworth said that though a definite diagnosis during life of infective endocarditis could only be made in the presence of three phenomena—a heart-murmur, fever, and infective emboli—and this endocarditis could only be held to be definitely due to streptococci when examination of the blood proved their presence; yet insomuch as this form of endocarditis was in the majority of cases due to these particular micro-organisms and there was no evidence that the injection of anti-streptococcic serum ever did harm, it was permissible for one to use this method of treatment in cases where the evidence was incomplete. He had found in several cases of rheumatic fever with endocarditis, where the fever continued after the arthritis had subsided and there was no lesion other than the cardiac one to account for the fever, that an injection of anti-streptococcic serum had caused a rapid disappearance of the fever, whilst apparently the cardiac mischief did not subsequently further advance.—Mr. Roger Williams mentioned that in certain cases of acute mastitis with septic complications the latter had been found to be due to the presence of staphylococci, which some pathologists thought were harmless, and he asked whether any analogous condition was found in ulcerative endocarditis.

### ACHONDROPLASIA.[1]

BY

Charles E. S. Flemming, M.R.C.S., L.R.C.P.

